# Potential of Fluoride-Containing Zinc Oxide and Copper Oxide Nanocomposites on Dentin Bonding Ability

**DOI:** 10.3390/nano12081291

**Published:** 2022-04-11

**Authors:** Bayarchimeg Altankhishig, Yasuhiro Matsuda, Futami Nagano-Takebe, Katsushi Okuyama, Hiroko Yamamoto, Masahiko Sakurai, Katsuaki Naito, Mikako Hayashi, Hidehiko Sano, Sharanbir K. Sidhu, Takashi Saito

**Affiliations:** 1 Division of Clinical Cariology and Endodontology, Department of Oral Rehabilitation, School of Dentistry, Health Sciences University of Hokkaido, Hokkaido 061-0293, Japan; bayarchimeg@hoku-iryo-u.ac.jp (B.A.); e19pl@hoku-iryo-u.ac.jp (M.S.); t-saito@hoku-iryo-u.ac.jp (T.S.); 2 Department of Dental Materials Science, School of Dentistry, Health Sciences University of Hokkaido, Hokkaido 061-0293, Japan; nagano23@hoku-iryo-u.ac.jp; 3 Department of Dental Materials Science, Asahi University School of Dentistry, Mizuho 501-0296, Japan; katsu@dent.asahi-u.ac.jp; 4 Department of Restorative Dentistry and Endodontology, Osaka University Graduate School of Dentistry, Suita 501-0296, Japan; yhiroko@dent.osaka-u.ac.jp (H.Y.); naito@dent.osaka-u.ac.jp (K.N.); mikarin@dent.osaka-u.ac.jp (M.H.); 5 Department of Restorative Dentistry, Hokkaido University Graduate School of Dental Medicine, Sapporo 060-8586, Japan; sano@den.hokudai.ac.jp; 6 Oral Bioengineering, Institute of Dentistry, Queen Mary University of London, London E1 4NS, UK; s.k.sidhu@qmul.ac.uk

**Keywords:** nanocomposite, antimicrobial, bioactive materials, microtensile bond strength, trace element

## Abstract

Despite recent advances in bonding restorations, which are the basis of restorative dentistry, secondary caries are still able to form. Previously, a novel fluoride-containing zinc and copper (ZCF) nanocomposite was introduced to prevent the formation of caries due to its antibacterial activity. In this study, we studied the impact of ZCF nanoparticles on the adhesive strength of bonding restorations through micro-tensile bond strength (µTBS) testing. The impact of antibacterial and matrix metalloproteinase (MMP) inhibitors on the nanoparticles was also examined. The nanocomposites were prepared using a simple one-step homogeneous co-precipitation method at a low temperature. A self-etch adhesive was applied to 10 extracted caries-free human molars with (test group) and without (control group) the ZCF nanoparticles. This was followed by composite resin build-up and µTBS testing, MMP activity assays, and evaluation of the antibacterial effects. The results showed no significant differences in the µTBS between the ZCF and the control groups. However, the ZCF exhibited a significant inhibitory effect against MMP-2, MMP-8, and MMP-9, in addition to an antibacterial effect on Streptococcus mutans. Therefore, the present study demonstrated that the addition of ZCF nanoparticles to adhesive systems can result in MMP inhibition and antibacterial action while maintaining the mechanical properties of the bonding restorations.

## 1. Introduction

Composite resins are widely used in clinical restorative dentistry applications because of their various desirable properties, such as good esthetics and high strength [1]. In particular, the minimally invasive nature of composite resin restorations was enhanced by improving the quality of the adhesive materials, and these resins yielded comparable clinical results to glass-ionomer cement, which possesses relatively inferior physical properties. However, failure of the restoration and the formation of secondary caries related to the composite resins still occur. In addition, the long-term clinical success of adhesive restorations depends on the long-term stability of the adhesive system in the dentin matrix [1]. Similarly, the prevention of dentin degradation is also considered an essential factor to ensure stable long-term adhesion, especially in cases where the lesion is present primarily in the dentin component, such as in root surface caries [2]. Since the progression of dentin caries is caused by demineralization upon the acidic and enzymatic degradation of collagen, the inhibition of enzymes, as well as enzyme-producing bacteria, is of particular interest. The development of antimicrobial and enzyme inhibitory materials that do not affect the mechanical properties and durability of adhesives is therefore necessary.

It was reported that zinc inhibits dentin demineralization while also promoting dentin remineralization. In addition, it prevents dental caries by exerting antibacterial effects on oral bacteria [3,4,5]. The matrix metalloproteinases (MMPs) are enzymes that degrade collagen fibers and require zinc as a cofactor. Although zinc is one of the most commonly used elements in dental materials and in mouth rinses, it acts as an inhibitor of MMPs at high concentrations [6,7]. Since biopolymers, such as collagen, are key to biomineralization [8], zinc promotes hard-tissue mineralization due to the fact that it protects collagen from the MMPs. Furthermore, the presence of zinc may protect any seed crystals present on collagen fibrils to allow subsequent dentin remineralization [9]. It is also well known that metals such as copper exhibit high antibacterial activities at low concentrations [10], and several studies have found that copper nanoparticles can inhibit MMPs while also activating the secretion of tissue inhibitors of MMPs [11,12]. Moreover, it was reported that Zn–CuO nanocomposites are less embryotoxic than zinc oxide (ZnO) and copper oxide (CuO) nanoparticles [11], and so they could potentially be considered safer antimicrobial metal nanocomposites.

In recent studies, copper nanoparticles were shown to induce antibacterial effects in adhesive bonding without reducing the mechanical properties of the bonding itself [13,14]. In addition, a combination of ZnO and CuO nanoparticles in an adhesive was found to exhibit antimicrobial and anti-MMP activities without affecting the bond strength [15]. Since fluorine is widely used to enhance the remineralization of dentin and enamel, Matsuda et al. previously introduced novel fluoride-containing ZnO and CuO nanocomposites (ZCFs), which they discovered exhibited strong antimicrobial properties [16]. However, to date, the incorporation of different combinations of ZCFs into adhesive systems has yet to be investigated and evaluated in detail.

Thus, we herein report our evaluation of the effects of ZCFs in dentin adhesive on the dentin bond strength using microtensile bond strength (μTBS) testing, which was widely used for evaluating the bonding abilities of dentin bonding systems. The µTBS test is preferred for evaluating adhesion to dentin because it uses smaller specimens, thereby resulting in fewer internal defects and more uniform stress distribution at the interface compared to macrotensile bond strength testing [17]. Subsequently, the anti-MMP activities and antibacterial effects of the ZCF systems are also examined.

## 2. Materials and Methods

### 2.1. Preparation and Observation of the Nanocomposites

#### 2.1.1. Preparation of the Fluoride-Containing ZnO and CuO Nanocomposites (ZCFs)

**Figure 1:**
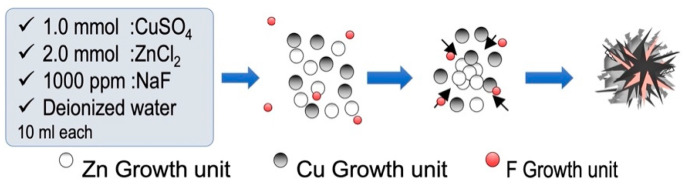
Schematic representation of ZCF nanocomposite synthesis.

The ZCFs were prepared according to a previous study [16]. Initially, equal volumes of ZnCl_2_ (2.0 mmol), CuSO_4_∙5H_2_O (1.0 mmol), NaF (1000 ppm), and NaOH (10.0 mmol) were mixed and maintained static at 80 °C for 24 h. The mixture was then allowed to cool to ~25 °C. The cooled solution was then subjected to centrifugation, washed several times with distilled water and absolute alcohol to remove any residues, and dried at 80 °C for 12 h (Figure 1).

#### 2.1.2. Scanning Electron Microscopy (SEM) Observations of the ZCF Nanocomposites

The prepared ZCF samples were mounted on an aluminum stub with an uncoated carbon tape and coated with palladium prior to scanning electron microscopy (SEM) observations. The particle morphologies and size distributions were analyzed at magnifications of 60 and 110 K using an S-4800 (Hitachi, Ibaraki, Japan) microscope operating at 5 kV. The energy dispersive X-ray spectra (EDS) of the samples were recorded using an S-2380N system (Hitachi, Ibaraki, Japan) equipped with a Genesis G4000 detector (EDAX, Tokyo, Japan) at 15 kV. The resulting spectra were used to determine the mineral and particle compositions. The elemental weight analysis (%) of the samples was carried out according to the ZAF correction method. 

#### 2.1.3. X-ray Photoelectron Spectroscopy (XPS)

The X-ray photoelectron spectroscopy (XPS) measurements were conducted using an ESCA-850 instrument (Shimazu Co. Ltd., Kyoto, Japan) with Al Kα radiation (1486.6 eV). All measurements were carried out under an ultra-high vacuum at 2 × 10^−6^ Pa. The binding energies of the measured photoelectron peaks were calibrated according to the C 1s peak of the hydrocarbon contamination at a binding energy of 285.0 eV. The experimental data were processed using the ESCA-850 spectrometer software (Shimazu Co. Ltd., Kyoto, Japan).

#### 2.1.4. Determining Fluoride Concentration Using PIGE and PIXE 

The fluorine content of each sample was analyzed using the proton-induced gamma-ray emission (PIGE) technique at the Takasaki Ion Accelerators for Advanced Radiation Application (TIARA) [18]. In-air micro-PIGE/PIXE (proton-induced X-ray emission) analysis was performed as described previously [19]. More specifically, the ZCF nanocomposite was attached directly to the window of an ion microbeam system, wherein a 3.0 MeV proton beam bombards the sample under ambient air conditions. The diameter of the beam spot was approximately 1 µm and the beam current was 100 pA. The maximum scanned area was 1000 µm^2^. A nuclear reaction [19F (*p*, αγ)160] was used for F estimation, wherein gamma rays were detected using an 81 cm^2^ sodium iodide detector placed at a distance of 5 mm behind the sample. Micro-PIXE was simultaneously performed using a silicon lithium detector in vacuum to estimate the Ca concentration. 

### 2.2. Effect of Nanocomposites on Dentin Bonding Ability

#### 2.2.1. Specimen Preparation and Bonding Procedures

This study was approved by the Ethics Committee of the Graduate School of Dentistry, Health Sciences University of Hokkaido, Japan (approval no. 47). The study protocol was approved by the Ethics committee of the Health Sciences University. 

**Figure 2:**
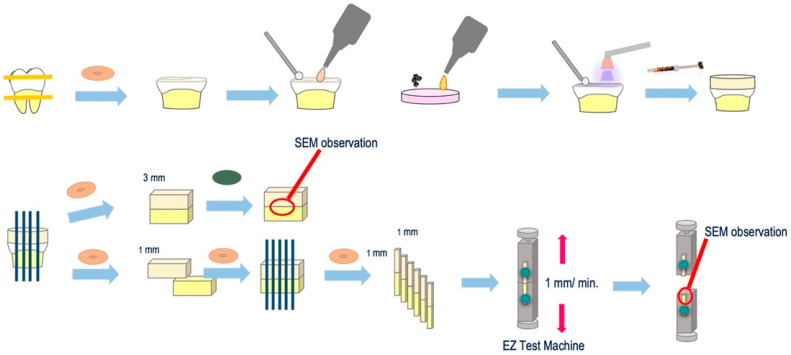
Experimental procedure for the micro-shear strength test.

The experimental procedure for µTBS test specimen preparation is outlined in Figure 2. More specifically, 10 caries-free, freshly extracted, human third molars were collected through informed consent from patients. The teeth were disinfected with 0.5% chloramine-T and used within three months of extraction. The occlusal enamel was cut to obtain a flat dentin surface through the use of a low-speed diamond saw (Isomet, Buehler, IL, USA) under running water. The dentin surface was ground using 600 grit silicon carbide paper (Carbimet Discs, Buehler, IL, USA) for 20 s under running water to create a smear layer. A self-etch adhesive primer (Clearfil SE Bond; Kuraray, Japan) was applied to the dry dentin surface for 20 s, followed by a gentle airflow. The 5 wt% ZCF powder was then added to the adhesive (Clearfil SE Bond, Kuraray, Japan), applied to the dentin surface for 20 s, gently air-dried, and light-cured for 10 s. In the control group, the bonding adhesive (without the ZCF powder) was applied according to the manufacturer’s instructions. After the bonding procedure, a composite resin (Clearfil AP-X, Kuraray, Japan) build-up (5 mm high) was incrementally performed, and after each increment, the sample was light-cured for 40 s. Subsequently, the teeth were stored in distilled water and incubated at 37 °C for 24 h (Figure 2). 

#### 2.2.2. Microtensile Bond Strength (µTBS) Testing

After incubation, all specimens were cut using a low-speed diamond saw (Isomet, Buehler, IL, USA). As a result, the cuts were perpendicular to the dentin surface at the bonding to obtain 1 mm square poles. One sample was cut to a thickness of 3 mm and polished to form a mirror surface for analyzing the resin–dentin interface by SEM (S-3500N, Hitachi, Japan). The remaining samples were sectioned into a beam shape (1.0 mm^2^ cross-section). Each stick was attached to a jig device for the µTBS test with cyanoacrylate resin (Model Repair II Blue, Dentsply Sirona, USA) and subjected to a tensile force in a universal testing machine (EZ test machine, Shimadzu, Japan) at a crosshead speed of 1.0 mm/min. The fractured surfaces were observed using SEM and classified as cohesive failure in the composite resin (CR), cohesive failure in the adhesive (A), failure at the bond–dentin interface (I), or cohesive failure in the dentin (CD).

### 2.3. Ion Release from ZCF

**Figure 3:**
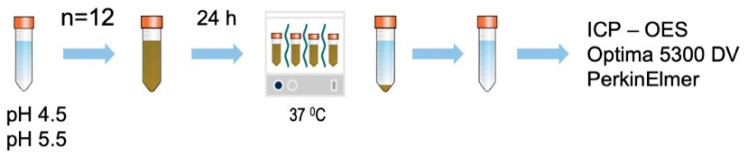
Schematic representation showing preparation of the samples for the ICP-OES measurements of ZCF to determine the degree of ion release.

Acetate buffer was freshly prepared and titrated to the desired pH of 4.5 and 5.5 acetic acid–sodium acetate buffer solution (FUJIFILM Wako chemical, Japan). The prepared ZCF nanocomposite powders were used to prepare 0.1% suspensions in the acetate buffers (10 mL, 12 tubes, pH 4.5 or 5.5) and maintained at 37 °C for 24 h (Figure 3). Subsequently, the products were separated by centrifugation, and the zinc and copper contents of the solution were analyzed using inductively coupled plasma-optical emission spectrometry (Optima 5300 DV PerkinElmer).

### 2.4. Anti-MMP Activity Tests

To conduct the anti-MMP activity test, the ZCF suspension was adjusted to a concentration of 0.2 mg/mL in distilled water. The supernatant from the dispersion of each nanocomposite was used for the purpose of testing. Following the manufacturer’s recommendations, recombinant MMP-2, MMP-8, and MMP-9 were employed in the MMP fluorometric assay kits (SensoLyte assay kits, AnaSpec, Fremont, CA, USA). The four nanocomposites and active MMPs (pre-incubated with 1 mM of amino-phenyl mercuric acetate) were mixed with the samples in 96-well plates. The 5-FAM/QXLTM 520 fluorescence resonance energy transfer peptide substrate assay buffer was then added to each 96-well plate and incubated for 1 h. The fluorescence intensity (relative fluorescence unit (RFU)) was measured using a LightCycler^®^96 fluorescence analyzer (Roche, Germany). The diluted active MMPs were used as positive controls.

### 2.5. Antibacterial Test

*Streptococcus mutans* (JCM 5705) was cultured on MS agar plates at 37 °C for 48 h. After this time, the colonies were collected and cultured in brain heart infusion (BHI) medium (10 mL), then incubated at 37 °C for 24 h. A bacterial (OD1) absorbance solution with PBS from cultured bacteria was prepared at a wavelength of 600 nm and was used in all experiments. Two resin discs (Unifast III, GC) were prepared using different concentrations of ZCF (i.e., 1.25 and 2.5%) of ZCF. Plain resin discs were used as controls. Subsequently, BHI medium (1950 µL, containing 1% sucrose) and the bacterial suspension (50 µL) were added to a single disc in each well and incubated at 37 °C for 48 h. The BHI medium was changed every 24 h. The number of bacteria was determined after 48 h of incubation. All experiments and controls were tested in quintuplicate (n = 5).

### 2.6. Statistical Analyses

All statistical analyses were performed using SPSS Statistics for Mac, Version 23.0 (IBM SPSS Statistics for Mac, Version 23.0. Armonk, NY: IBM Corp). The data were analyzed statistically using Student’s *t*-test (*p* < 0.05) to compare the control and ZCF groups.

## 3. Results

### 3.1. SEM Observations of the Nanocomposite

**Figure 4:**
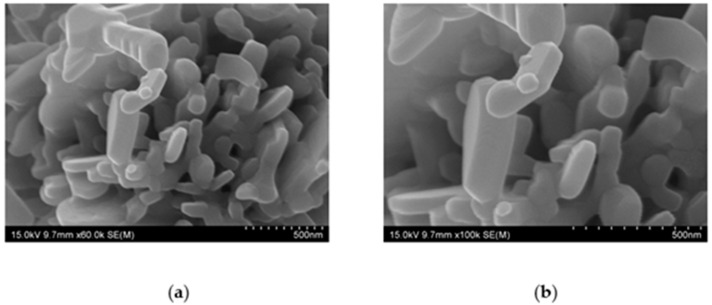
SEM images of the prepared fluorine-containing ZnO–CuO (ZCF) nanocomposites at (**a**) ×60 K and (**b**) ×100 K magnifications.

The morphological and elemental compositions of the ZCF nanocomposite were determined by means of SEM analysis. Figure 4 shows the SEM micrographs of the nanocomposite, which indicate that the particles were firmly attached and agglomerated. The average size of the ZCF nanocomposites, which exhibited a square crystal structure, was approximately 100 nm (Figure 4). 

**Figure 5:**
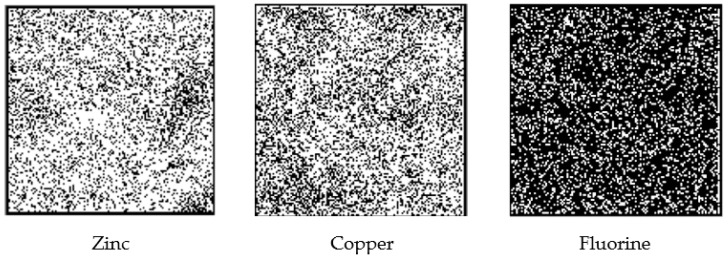
Elemental PIXE maps (zinc and copper) and PIGE map (fluorine). The white dots in the maps (250 µm × 250 µm area) represent the PIXE or PIGE signals from zinc, copper, and fluorine, respectively.

The quantitative analysis determined using the ZAF method yielded EDS results, which showed weight ratios of Zn: 40.9 ± 3.3%; Cu: 47.6 ± 2.0%; F: 6.0 ± 1.4%, and Cl: 5.1 ± 1.8%. The quantitative fluorine analysis by PIGE showed 2499.0 ± 98.5 ppm of fluorine (Figure 5).

**Figure 6:**
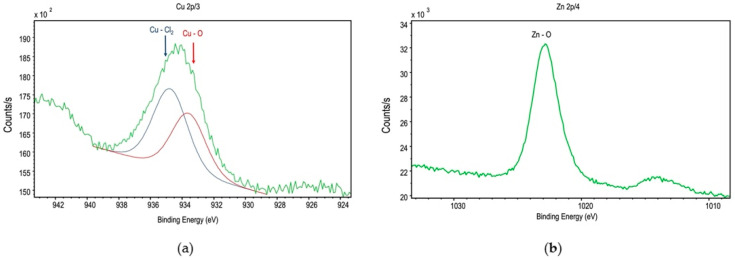
XPS spectra of ZCF: (**a**) Cu 2p and (**b**) Zn 2p.

The XPS can provide sensitive information regarding the surface chemical compositions of materials. In the case of ZCF, Cu peaks were observed in the XPS spectrum (Figure 6a), which corresponded to Cu2p 3/2. In addition, fused spectra were obtained using the ESCA-850 spectrometer software, along with peaks corresponding to the Cu–O and Cu–Cl_2_ binding energies. The presence of CuO indicates that the copper present on the surface region was oxidized easily at ~25 °C. In addition, the presence of CuCl_2_ was attributed to the use of ZnCl_2_ during the synthetic process. Furthermore, the peaks corresponding to the Zn–O binding energy were observed (Figure 6b). Moreover, the quantitative XPS analysis yielded a composition of 24.0% Cu, 60.6% Zn, 5.8% Cl, and 9.6% Na. Because the zinc ratio obtained using XPS was higher than that determined by EDS, it appeared that the ZCF possessed a high surface area.

### 3.2. µTBS Tests

**Figure 7:**
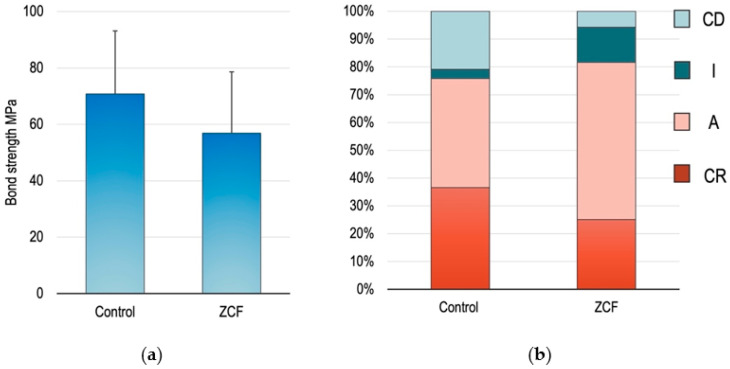
(**a**) Descriptive statistics of the different groups assigned in the microtensile bond strength tests. The mean and SD are shown. (**b**) Comparison of fracture types between the different groups.

The mean values and standard deviations of the µTBS test results are presented in Figure 7, wherein no significant differences were observed in the bond strengths between the ZCF group (66.9 ± 7.4 MPa) and the control group (77.1 ± 13.8 MPa). The fractured surfaces were divided into four groups, as described above, namely the CR, A, I, and CD groups. The fracture types of the CR and A are defined as failures occurring in the material that maintains the dentin–bond interface. In contrast, I and CD are defined as failures occurring at the dentin–bond interface. Based on these failure modes and results, it was clear that the amount of adhesive remaining in the dentin was higher in the experimental group than in the control group, indicating that the hybrid layer (made of collagen fiber of dentin and bonding agent) present between dentin and the adhesive increased with the addition of ZCF.

**Figure 8:**
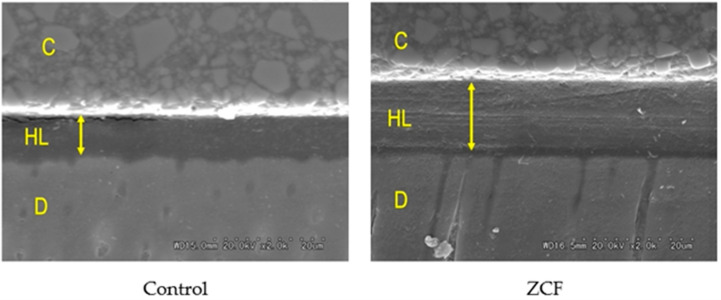
SEM observations of the resin–dentin interface. C = resin composition, HL = hybrid layer, D = dentin. The arrow indicates the thickness of the hybrid layer.

Furthermore, SEM observations of the resin–dentin interface showed that the hybrid layer of ZCF was thicker than that of the control group (Figure 8). The concentrations of zinc and copper ions on the hybrid layer were 2.27 ± 1.82 and 0.76 ± 0.50 ppm, respectively, from EDX analysis. The EDX data showed very low concentrations of zinc and copper than 5% of wt%. This would have resulted in a lower concentration in the EDX analysis due to a smaller volume ratio compared to the low-density bonding material.

### 3.3. Ion Release from ZCF

The concentrations of zinc and copper ions released from the ZCF in acetate buffer were then determined and the results are presented in Table 1 (*t*-test, *p* < 0.05). From these results, it can be seen that the number of ions released at pH 4.5 was significantly greater than that at pH 5.5. 

### 3.4. Anti-MMP Tests

**Figure 9:**
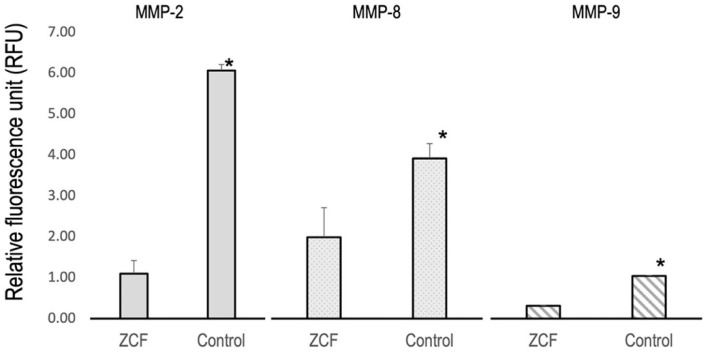
Assay results for the inhibition of MMP–2, MMP–8, and MMP–9 by ZCF using diluted active MMP as a positive control (* *p* < 0.05).

The activity levels of MMP-2, MMP-8, and MMP-9 are shown in Figure 9, wherein it can be seen that the MMP activities were significantly lower in the ZCF group than in the control group (*p* < 0.05), thereby suggesting that the released zinc and copper ions exhibited MMP inhibitory effects. As mentioned above, MMP inhibitors were reported to inhibit dentin demineralization, and so importantly, ZCF also appears to exhibit the same inhibitory effect. 

### 3.5. Antibacterial Tests

**Figure 10:**
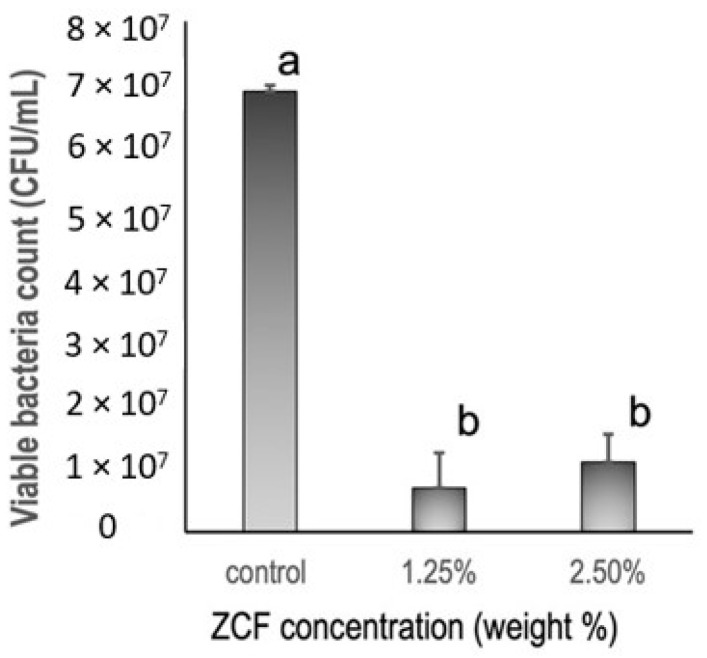
Antibacterial activities of resin discs containing different concentrations of the ZCF nanocomposites and the control, as determined by CFU analysis.

Based on the results of the antibacterial tests (Figure 10), it was apparent that the ZCF groups showed significant differences from the control group. More specifically, the control group gave 6.9 × 10^7^ ± 3.3 × 10^7^ CFUs/mL of *Streptococcus mutans* growth, while the ZCF groups at concentrations of 1.25 and 2.5 wt% gave values of 6.9 × 10^6^ ± 7.2 × 10^6^ and 1.1 × 10^7^ ± 8.3 × 10^6^ CFUs/mL of bacterial growth, respectively. 

## 4. Discussion

Following the preparation of the desired nanocomposites, their sizes ranged from tens to hundreds of nanometers, and their zinc and copper concentrations were approximately equal. The combination of ZnO and CuO nanocomposites has been used in various fields because of its better results compared to individual components (ZnO or CuO) [20]. Many researchers have prepared ZnO and CuO nanocomposites using various methods such as sol-gel [21], chemical vapor deposition [22], co-precipitation [23], thermal decomposition [24], and complex-directed hybridization, and heating brass in the air [25]. The co-precipitation method was used to produce superfine insoluble compounds based on a combination of metal solutions, wherein an increase in the solution pH can cause the metal ions to oxidize [26]. Because these oxides are more stable compared to the corresponding chlorides and fluorides, the latter is easily removed upon washing with distilled water. Ultimately, this led to the preparation of nanocomposites that were mainly composed of zinc oxide and copper oxide. It was observed that although both copper and zinc leached into the solution, the zinc leaching was dominant, and greater degrees of leaching were observed at a lower solution pH (i.e., 4.5 c.f., 5.5). This can be attributed to the stronger ionization tendency of zinc compared to copper, and thus, on the ZCF surface, zinc is more likely to become ionized and leach into the solution. In contrast, copper exists as CuO and CuCl_2_ on the ZCF surface, and thus, chloride ions are also released into the solution, which also exhibits an antibacterial effect. It should also be noted here that the ZnO–CuO nanocomposite exhibits a photocatalytic activity, and it generates free radicals [23], both of which result in an enhanced antibacterial effect.

Since the quantitative analysis of fluorine in hard tissue is complicated using specific X-ray analyses, we developed and employed an analytical method based on the use of specific gamma rays [16,27,28,29,30] to analyze the fluorine contents of the samples. It should be noted that the differences in fluorine values obtained for the EDS and the in-air micro-PIXE/PIGE techniques can be attributed to the different sensitivities and specificities of the analytical methods. Scanning Electron Microscope Energy Dispersive X-ray Spectroscopy (SEM-EDS) is used bombardment of the specimen by electrons to induce characteristic X-rays from the sample, which are then detected using wavelength-dispersive spectrometers (EDS). The characteristic X-ray energy of each element depends on the element size, The characteristic X-ray energy of a light element is small. Therefore, it is not suitable for the measurement of a light elements such as fluorine, boron, lithium. The PIGE procedure detects the characteristic gamma-ray energy, and it does not depend on the element size, so it is suitable for measuring light elements.

As mentioned above, MMP inhibitors were reported to inhibit dentin demineralization, and importantly, our results indicate that the ZCF nanocomposites also exhibit this inhibitory effect. As a result, a re-mineralizing effect is expected, wherein biomacromolecules form mesocrystal cores to induce systematic mineralization [8], and this process is believed to be promoted by the collagen that is preserved by the MMP inhibitors. Therefore, the protection of collagen from MMPs is key to reducing the degradation of dentin, and enhancing remineralization, so the MMP inhibitory effects of the ZCF nanocomposites are of particular importance. Although several previous studies have demonstrated the MMP inhibitory properties of zinc [31] and copper [12], no study has described the MMP inhibitory potential of nanocomposites containing both ions. In the current study, we demonstrated that ZCF inhibited the expression of MMP-2, MMP-8, and MMP-9. 

Furthermore, although fluorine is one of the most critical elements in the prevention and clinical treatment of caries, most nanocomposites do not tend to contain light elements such as fluorine. However, the co-precipitation method employed herein for nanocomposite preparation is advantageous when considering the addition of light elements. Because the light element is easy it uses solutions of zinc, copper, and fluorine at low temperatures. As a result, the obtained ZCF contains nearly 3000 ppm fluorine, which is higher than the fluorine concentration that is known to reversibly inhibit the activity of MMPs [32,33]. More specifically, according to Kato et al., fluorine concentrations >2500 ppm reversibly inhibit MMPs, whereas fluorine concentrations >5000 ppm irreversibly inhibit MMPs. Among the existing dental materials, fluorine-releasing glass-ionomer cement is also known to exhibit antibacterial properties [32]. 

The aim of zinc and copper incorporation into the adhesive formulations was to impart antimicrobial properties. To date, several studies have demonstrated the antimicrobial properties of zinc nanoparticles [34] and copper nanoparticles [35] when evaluated alone or when dental adhesives were doped with both zinc and copper nanoparticles [36]. For example, our previous study showed that the antibacterial effect of a combination of zinc and copper nanocomposites inhibited the growth of *Streptococcus mutans* [16]. Importantly, Zn–CuO nanocomposites are reported to be less embryotoxic than ZnO and CuO nanoparticles alone, and so they are preferrable for use in a physiological system [10]. Our data showed that resin discs that contained ZCF inhibited *Streptococcus mutans* growth. Since various types of resins are used in dentistry, the antibacterial ZCFs are expected to be widely applicable in clinical practice. Moreover, the increased ion release in acidic environments is important due the acidic environment of dental caries, which are caused by demineralization of the tooth structure by the bacterially-produced organic acids adhering to the dentin surface. The ZCF nanocomposites are therefore expected to be beneficial in inhibiting the formation of caries.

In terms of their current clinical applications, zinc is commonly used as a commercial dental product because of its antibacterial effects; some mouthwashes contain zinc chloride [37], and additionally, zinc gluconate was reported to significantly reduce the duration of symptoms of the common cold [38]. In addition, copper is considered a potent inhibitor of MMPs in human dentin [11], and this element is also used in the crosslinking of collagen in bone [39], which has a similar structure to dentin. It was assumed that the copper nanoparticles adhere to DNA to form crosslinks within and between nucleic acid chains, thereby disrupting the helical structure of DNA and inhibiting bacterial growth, while also hindering some biochemical processes taking place inside the bacterial cells. The combination of metal nanoparticles (e.g., copper and silver) could therefore yield a complete bactericidal effect against mixed bacterial populations [40].

In this study, the dentin bond strength in the ZCF group was similar to that in the control group. It should be noted here that a recent systematic review and meta-analysis stated that MMP inhibitors, such as the ZCFs prepared herein, do not have a significant impact on the short-term bond strength but they affect the long-term bond strength [41]. The ZCF that was developed also showed MMP inhibition, and thus, the dentin bond strength was similar in the ZCF group. Regarding the failure mode of the bonded surface, the ZCF group showed a minor CD failure and an I failure, and the bond interface tended to be more protected than that of the control. However, many fracture modes were also observed within the bonding agent. These observations were attributed to the weak interactions between the nanocomposite and the resin monomer in the bonding agent. Furthermore, it was previously reported that a thicker bonding layer in the self-etch adhesive system, the higher the bond strength [42]. In this study, the thickness of the hybrid layer in the ZCF group is greater than that of the control. It should also be highlighted that the addition of ZCF to adhesive systems did not reduce the µTBS of the original materials.

## 5. Conclusions

This study investigated the properties of a novel fluoride-containing zinc and copper nanocomposite upon its combination with an adhesive dental material. It was found that the addition of zinc and copper nanocomposites to the self-etch adhesive system imparted anti-MMP (matrix metalloproteinase) properties without having any negative influence on the mechanical properties of the material, thereby improving the quality of the resin–dentin interface. In addition, the added resin exhibited antibacterial properties, and so could help to inhibit the growth of caries. It was suggested that this novel nanocomposite could easily add new functions to adhesive dental materials. Further development and investigation of the nanocomposite, such as its ability to impart an improved long-term adhesiveness and strength to dentin, is expected in the future, and the results will be presented in due course.

## Figures and Tables

**Table 1:** Zinc and copper ion release in acetate buffer.

	Zinc (ppm)	Copper (ppm)
pH 4.5	568.7 ± 14.0 (a)	270.8 ± 7.2 (a)
pH 5.5	352.2 ± 13.2 (b)	2.4 ± 1.2 (b)

## Data Availability

Not applicable.
